# The genome sequence of a rove beetle, *Philonthus spinipes *Sharp, 1874

**DOI:** 10.12688/wellcomeopenres.23996.1

**Published:** 2025-04-25

**Authors:** Darren J. Mann, Liam M. Crowley, University of Oxford and Wytham Woods Genome Acquisition Lab, Darwin Tree of Life Barcoding collective, Wellcome Sanger Institute Tree of Life Management, Samples and Laboratory team, Wellcome Sanger Institute Scientific Operations: Sequencing Operations, Wellcome Sanger Institute Tree of Life Core Informatics team, Tree of Life Core Informatics collective, Darwin Tree of Life Consortium

**Affiliations:** 1 Oxford University Museum of Natural History, Oxford, England, UK; 2 University of Oxford, Oxford, England, UK

**Keywords:** Philonthus spinipes, beetle, genome sequence, chromosomal, Coleoptera

## Abstract

We present a genome assembly from a female *Philonthus spinipes* (rove beetle; Arthropoda; Insecta; Coleoptera; Staphylinidae). The genome sequence has a total length of 671.10 megabases. Most of the assembly is scaffolded into 25 chromosomal pseudomolecules, including the X sex chromosome. The mitochondrial genome has also been assembled and is 19.08 kilobases in length. Gene annotation of this assembly on Ensembl identified 30,004 protein-coding genes.

## Species taxonomy

Eukaryota; Opisthokonta; Metazoa; Eumetazoa; Bilateria; Protostomia; Ecdysozoa; Panarthropoda; Arthropoda; Mandibulata; Pancrustacea; Hexapoda; Insecta; Dicondylia; Pterygota; Neoptera; Endopterygota; Coleoptera; Polyphaga; Staphyliniformia; Staphylinoidea; Staphylinidae; Staphylininae group; Staphylininae; Staphylinini; *Philonthus; Philonthus spinipes* Sharp, 1874 (NCBI:txid878362).

## Background

*Philonthus spinipes* was first described from East Asia by David Sharp in 1874, and is now found across the Palaearctic region. Its original range is unclear due to its westward expansion in recent decades ( Schillhammer, 1999). It was first recorded in Europe in the 1980s, with subsequent reports from various countries, including Poland, where it occurs in both lowland and mountainous areas ( Burakowski *et al.*, 2000; Schulke & Uhlig, 1989a; Schulke & Uhlig, 1989b).

This species is a flying, epigeic beetle associated with decomposing organic matter, including dung, compost, carrion, and plant debris. It is a predator, with both larvae and adults feeding on small insects such as coprophagous beetles and flies ( Pawlowski, 2008). *Philonthus spinipes* is considered an adventive species, rapidly colonising new habitats, including high-altitude regions ( Laszlo, 2004). There is evidence that *P. spinipes* competes with native species, particularly *Philonthus nitidus*, which has declined in parts of central Europe ( Schillhammer, 1999).

Here we present a chromosome-level genome sequence for *Philonthus spinipes*, based on a female specimen from Wytham Woods, Oxfordshire, UK.

## Genome sequence report

### Sequencing data

**Figure 1.**
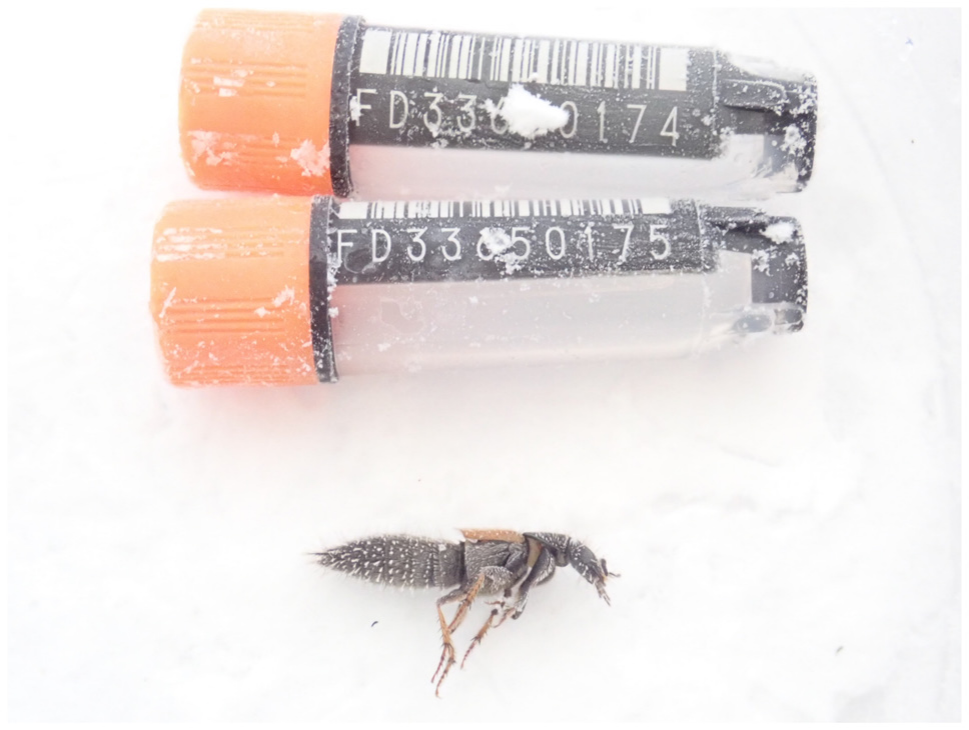
Photograph of the *Philonthus spinipes* (icPhiSpin1) specimen used for genome sequencing.

The genome of an adult female *Philonthus spinipes* ( Figure 1) was sequenced using Pacific Biosciences single-molecule HiFi long reads, generating a total of 26.96 Gb (gigabases) from 2.54 million reads, providing approximately 37-fold coverage. Primary assembly contigs were scaffolded with chromosome conformation Hi-C data, which produced 153.41 Gb from 1,015.96 million reads. Table 1 summarises the specimen and sequencing information.

**Table 1.** Specimen and sequencing data for *Philonthus spinipes*.

Project information			
**Study title**	Philonthus spinipes		
**Umbrella BioProject**	PRJEB61909		
**Species**	*Philonthus spinipes*		
**BioSample**	SAMEA112232682		
**NCBI taxonomy ID**	878362		
Specimen information			
**Technology**	**ToLID**	**BioSample accession**	**Organism part**
**PacBio long read sequencing**	icPhiSpin1	SAMEA112233159	head and thorax
**Hi-C sequencing**	icPhiSpin1	SAMEA112233159	head and thorax
**RNA sequencing**	icPhiSpin1	SAMEA112233160	abdomen
Sequencing information			
**Platform**	**Run accession**	**Read count**	**Base count (Gb)**
**Hi-C Illumina NovaSeq 6000**	ERR11439641	1.02e+09	153.41
**PacBio Sequel IIe**	ERR11435986	2.54e+06	26.96
**RNA Illumina NovaSeq X**	ERR12862075	6.16e+07	9.3

### Assembly statistics

The primary haplotype was assembled, and contigs corresponding to an alternate haplotype were also deposited in INSDC databases. The assembly was improved by manual curation, which corrected 67 missing joins or mis-joins and 13 haplotypic duplications, reducing the assembly length by 13.56% and the scaffold number by 98.96%, and increasing the scaffold N50 by 14.31%. The final assembly has a total length of 671.10 Mb in 30 sequence scaffolds with a scaffold N50 of 27.3 Mb and 149 gaps ( Table 2).

**Table 2.** Genome assembly data for *Philonthus spinipes*, icPhiSpin1.1.

Genome assembly		
Assembly name	icPhiSpin1.1	
Assembly accession	GCA_963082785.1	
*Accession of alternate haplotype*	*GCA_963082675.1*	
Span (Mb)	671.10	
Number of contigs	180	
Number of scaffolds	30	
Longest scaffold (Mb)	40.02	
Assembly metrics *		*Benchmark*
Contig N50 length (Mb)	8.2	*≥ 1 Mb*
Scaffold N50 length (Mb)	27.3	*= chromosome N50*
Consensus quality (QV)	66.3	*≥ 40*
*k*-mer completeness	Primary: 66.81%; alternate: 65.29%; combined: 98.13%.	*≥ 95%*
BUSCO **	C:99.2%[S:96.9%,D:2.2%], F:0.4%,M:0.5%,n:2,124	*S > 90%; D < 5%*
Percentage of assembly mapped to chromosomes	99.99%	*≥ 90%*
Sex chromosomes	X	*localised homologous pairs*
Organelles	Mitochondrial genome: 19.08 kb	*complete single alleles*
Genome annotation of assembly GCA_963082785.1 at Ensembl		
Number of protein-coding genes	30,004	
Number of gene transcripts	30,257	

* Assembly metric benchmarks are adapted from column VGP-2020 of “Table 1: Proposed standards and metrics for defining genome assembly quality” from Rhie *et al.* (2021). ** BUSCO scores based on the endopterygota_odb10 BUSCO set using version 5.3.2. C = complete [S = single copy, D = duplicated], F = fragmented, M = missing, n = number of orthologues in comparison. A full set of BUSCO scores is available at https://blobtoolkit.genomehubs.org/view/CAUJBH01/dataset/CAUJBH01/busco.

**Figure 2.**
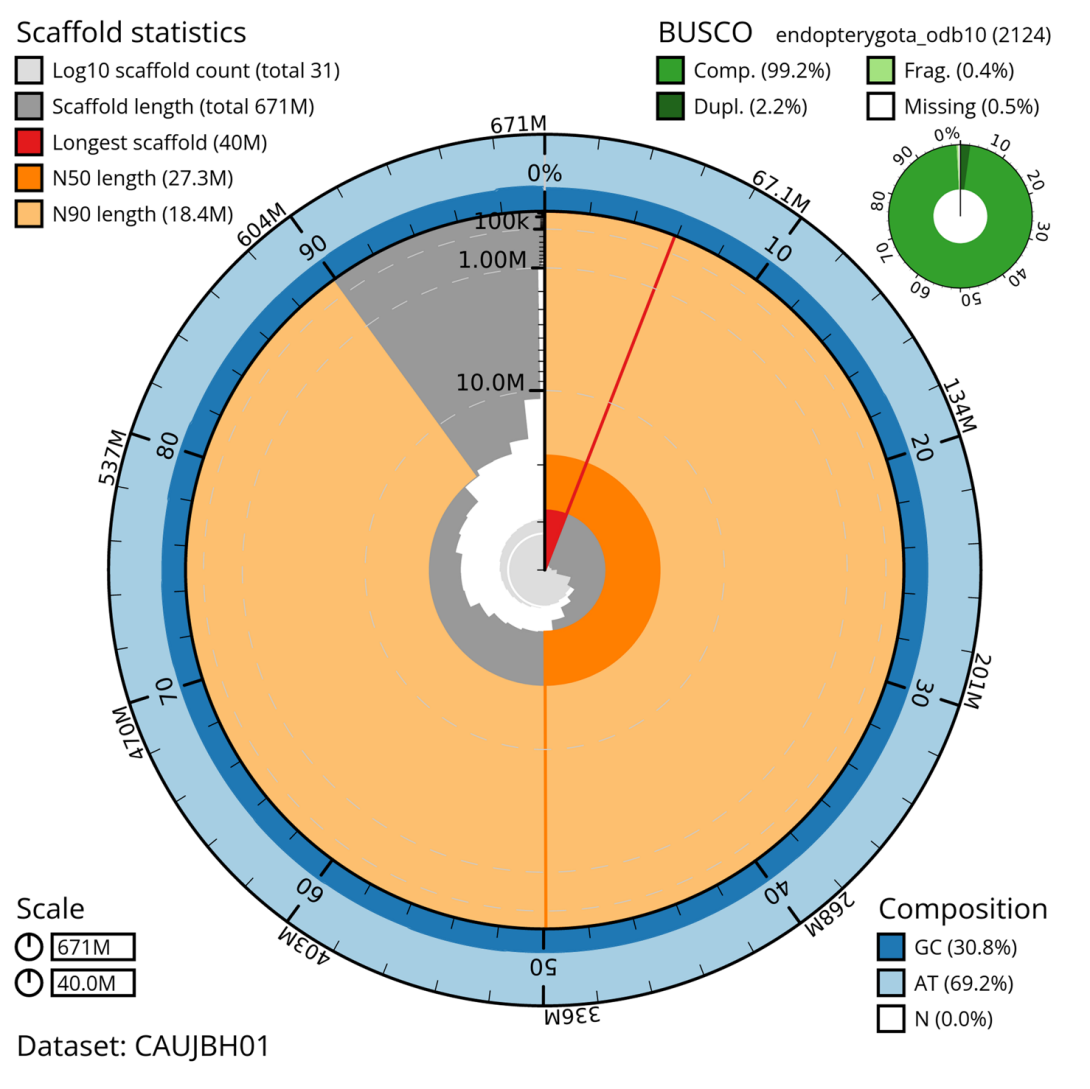
Genome assembly of *Philonthus spinipes*, icPhiSpin1.1: metrics. The BlobToolKit snail plot shows N50 metrics and BUSCO gene completeness. The circumference represents the length of the whole genome sequence, and the main plot is divided into 1,000 bins around the circumference. The outermost blue tracks display the distribution of GC, AT, and N percentages across the bins. Scaffolds are arranged clockwise from longest to shortest and are depicted in dark grey. The longest scaffold is indicated by the red arc, and the deeper orange and pale orange arcs represent the N50 and N90 lengths. A light grey spiral at the centre shows the cumulative scaffold count on a logarithmic scale. A summary of complete, fragmented, duplicated, and missing BUSCO genes in the endopterygota_odb10 set is presented at the top right. An interactive version of this figure is available at https://blobtoolkit.genomehubs.org/view/CAUJBH01/dataset/CAUJBH01/snail.

**Figure 3.**
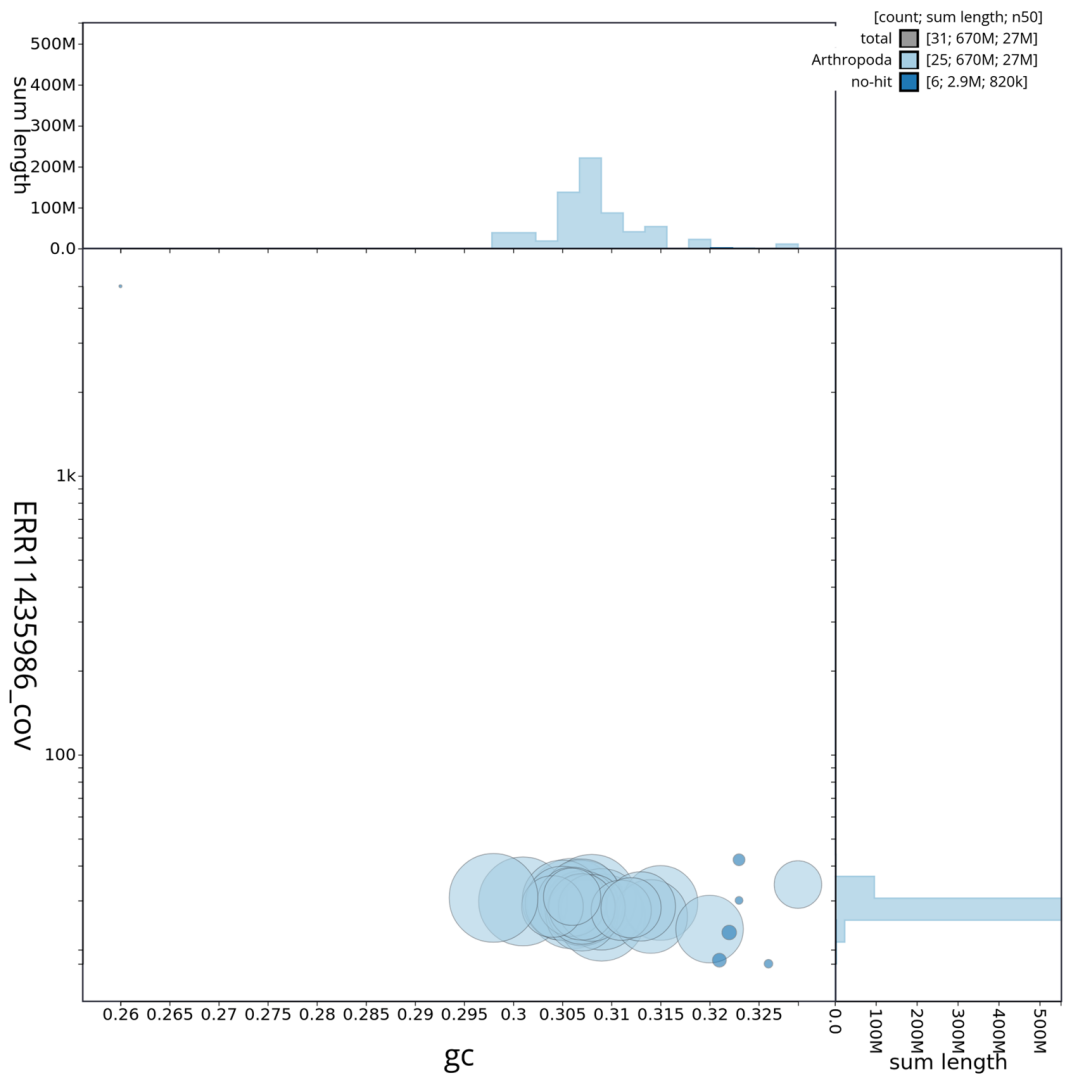
Genome assembly of *Philonthus spinipes*, icPhiSpin1.1: BlobToolKit GC-coverage plot. Sequences are coloured by phylum. Circles are sized in proportion to sequence length. Histograms show the distribution of sequence length sum along each axis. An interactive version of this figure is available at https://blobtoolkit.genomehubs.org/view/CAUJBH01/dataset/CAUJBH01/blob.

**Figure 4.**
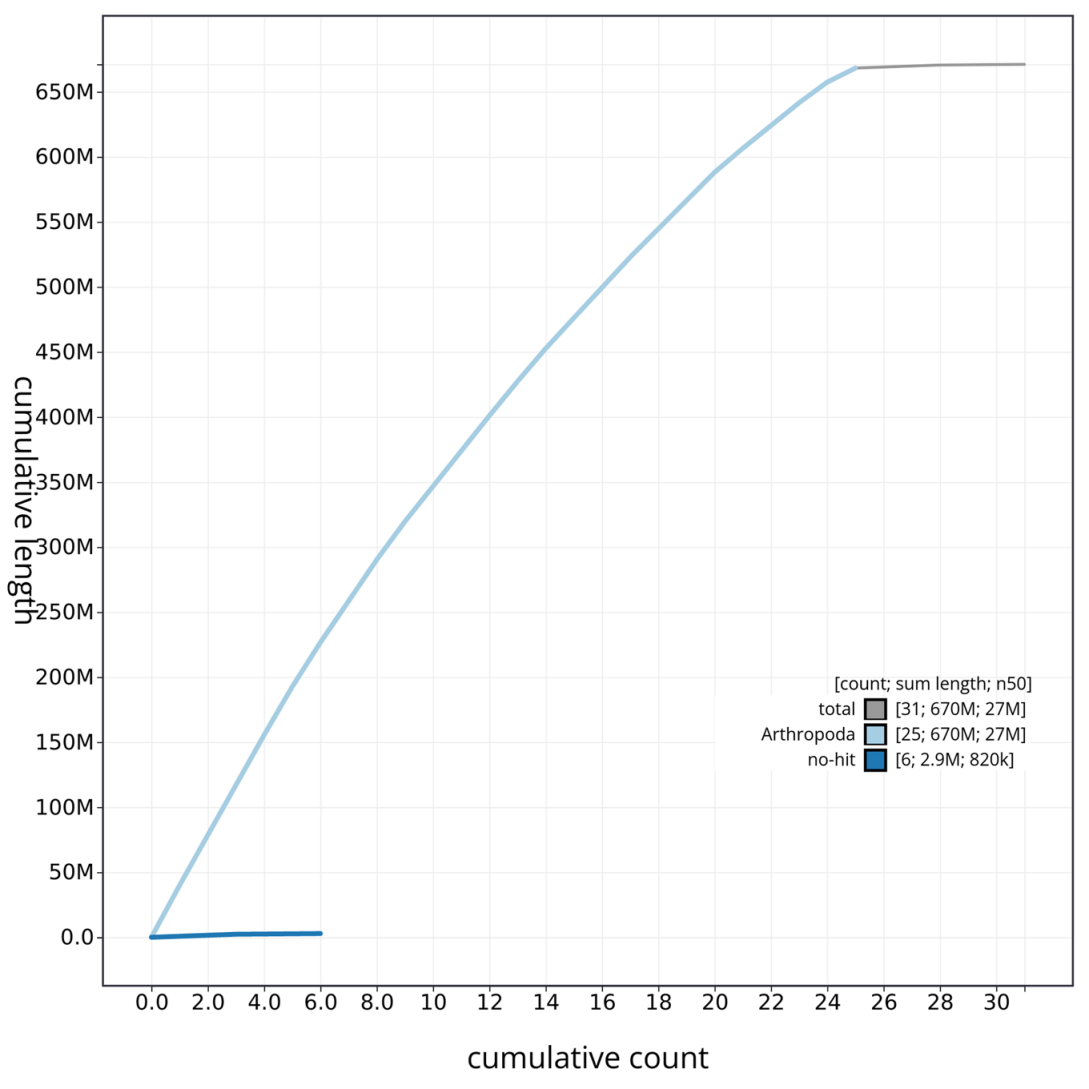
Genome assembly of *Philonthus spinipes* icPhiSpin1.1: BlobToolKit cumulative sequence plot. The grey line shows cumulative length for all sequences. Coloured lines show cumulative lengths of sequences assigned to each phylum using the buscogenes taxrule. An interactive version of this figure is available at https://blobtoolkit.genomehubs.org/view/CAUJBH01/dataset/CAUJBH01/cumulative.

**Figure 5.**
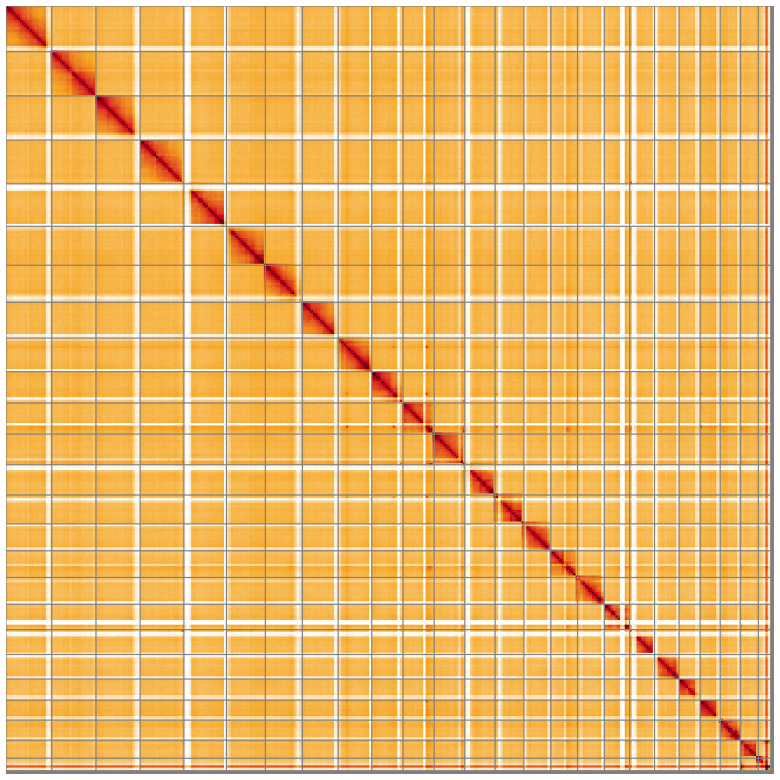
Genome assembly of *Philonthus spinipes* icPhiSpin1.1: Hi-C contact map of the icPhiSpin1.1 assembly, visualised using HiGlass. Chromosomes are shown in order of size from left to right and top to bottom. An interactive version of this figure may be viewed at https://genome-note-higlass.tol.sanger.ac.uk/l/?d=JfeiYCGcR-qCcodiB-XmVA.

The snail plot in Figure 2 provides a summary of the assembly statistics, while the distribution of assembly scaffolds on GC proportion and coverage is shown in Figure 3. The cumulative assembly plot in Figure 4 shows curves for subsets of scaffolds assigned to different phyla. Most of the assembly sequence (99.99%) was assigned to 25 chromosomal-level scaffolds, representing 24 autosomes and the X sex chromosome. Chromosome-scale scaffolds confirmed by the Hi-C data are named in order of size ( Figure 5; Table 3). Chromosome X was assigned based on synteny to the genome of *Neocrepidodera transversa* (GCA_963243735.1).

**Table 3.** Chromosomal pseudomolecules in the genome assembly of *Philonthus spinipes*, icPhiSpin1.

INSDC accession	Name	Length (Mb)	GC%
OY720299.1	1	40.02	30.5
OY720300.1	2	38.59	30.0
OY720302.1	3	38.29	31.0
OY720303.1	4	37.13	31.0
OY720304.1	5	34.04	30.5
OY720305.1	6	32.33	31.0
OY720306.1	7	31.28	30.5
OY720307.1	8	29.33	30.5
OY720308.1	9	27.35	30.5
OY720309.1	10	27.2	31.5
OY720310.1	11	26.82	30.5
OY720311.1	12	26.42	31.5
OY720312.1	13	25.22	30.5
OY720313.1	14	23.52	30.5
OY720314.1	15	23.35	31.5
OY720315.1	16	23.33	30.5
OY720316.1	17	22.17	32.0
OY720317.1	18	21.75	31.0
OY720318.1	19	21.41	30.5
OY720319.1	20	18.37	30.5
OY720320.1	21	17.55	31.0
OY720321.1	22	17.5	31.0
OY720322.1	23	15.84	30.5
OY720323.1	24	10.83	33.0
OY720301.1	X	38.57	30.0
OY720324.1	MT	0.02	27.0

The mitochondrial genome was also assembled and can be found as a contig within the multifasta file of the genome submission.

### Assembly quality metrics

The estimated Quality Value (QV) and *k*-mer completeness metrics, along with BUSCO completeness scores, were calculated for each haplotype and the combined assembly. The QV reflects the base-level accuracy of the assembly, while *k*-mer completeness indicates the proportion of expected *k*-mers identified in the assembly. BUSCO scores provide a measure of completeness based on benchmarking universal single-copy orthologues.

The estimated Quality Value (QV) of the final primary assembly is 66.3 (corresponding to an error rate of less than 1 per 1,000,000 bases). The *k*-mer recovery for the primary assembly is 66.81%, for the alternate assembly 65.29%, and for the combined primary and alternate assemblies 98.13%. BUSCO v5.3.2 analysis using the endopterygota_odb10 reference set ( *n* = 2,124) identified 99.2% of the expected gene set (single = 96.9%, duplicated = 2.2%).

## Genome annotation report

The *Philonthus spinipes* genome assembly (GCA_963082785.1) was annotated at the European Bioinformatics Institute (EBI) on Ensembl Rapid Release. The resulting annotation includes 30,257 transcribed mRNAs from 30,004 protein-coding genes ( Table 2; https://rapid.ensembl.org/Philonthus_spinipes_GCA_963082785.1/Info/Index). The average transcript length is 5,106.10. There are 1.01 coding transcripts per gene and 3.30 exons per transcript.

## Methods

### Sample acquisition and DNA barcoding

An adult female *Philonthus spinipes* (specimen ID Ox002479, ToLID icPhiSpin1) was collected from Wytham Woods, Oxfordshire, UK (latitude 51.78, longitude –1.32) on 2022-06-13 by potting. The specimen was collected by Darren Mann and Liam Crowley (University of Oxford), identified by Darren Mann and preserved on dry ice.

The initial identification was verified by an additional DNA barcoding process according to the framework developed by Twyford *et al*. (2024). A small sample was dissected from the specimens and stored in ethanol, while the remaining parts of the specimen were shipped on dry ice to the Wellcome Sanger Institute (WSI). The tissue was lysed, the COI marker region was amplified by PCR, and amplicons were sequenced and compared to the BOLD database, confirming the species identification ( Crowley *et al.*, 2023). Following whole genome sequence generation, the relevant DNA barcode region is also used alongside the initial barcoding data for sample tracking at the WSI ( Twyford *et al.*, 2024). The standard operating procedures for Darwin Tree of Life barcoding have been deposited on protocols.io ( Beasley *et al.*, 2023).

Metadata collection for samples adhered to the Darwin Tree of Life project standards described by Lawniczak *et al*. (2022).

### Nucleic acid extraction

The workflow for high molecular weight (HMW) DNA extraction at the Wellcome Sanger Institute (WSI) Tree of Life Core Laboratory includes a sequence of procedures: sample preparation; sample homogenisation, DNA extraction, fragmentation, and clean-up. Protocols developed by the WSI Tree of Life laboratory are publicly available on protocols.io ( Denton *et al.*, 2023b).

In sample preparation, the icPhiSpin1 sample was weighed and dissected on dry ice ( Jay *et al.*, 2023). Tissue from the head and thorax was homogenised using a PowerMasher II tissue disruptor ( Denton *et al.*, 2023a).

HMW DNA was extracted in the WSI Scientific Operations core using the Automated MagAttract v2 protocol ( Oatley *et al.*, 2023). The DNA was sheared into an average fragment size of 12–20 kb in a Megaruptor 3 system ( Bates *et al.*, 2023). Sheared DNA was purified by solid-phase reversible immobilisation, using AMPure PB beads to eliminate shorter fragments and concentrate the DNA ( Strickland *et al.*, 2023). The concentration of the sheared and purified DNA was assessed using a Nanodrop spectrophotometer and Qubit Fluorometer using the Qubit dsDNA High Sensitivity Assay kit. Fragment size distribution was evaluated by running the sample on the FemtoPulse system.

RNA was extracted from abdomen tissue of icPhiSpin1 in the Tree of Life Laboratory at the WSI using the RNA Extraction: Automated MagMax™ *mir*Vana protocol ( do Amaral *et al.*, 2023). The RNA concentration was assessed using a Nanodrop spectrophotometer and a Qubit Fluorometer using the Qubit RNA Broad-Range Assay kit. Analysis of the integrity of the RNA was done using the Agilent RNA 6000 Pico Kit and Eukaryotic Total RNA assay.

### Hi-C sample preparation

Tissue from the head and thorax tissue of icPhiSpin1 sample was processed for Hi-C sequencing at the WSI Scientific Operations core, using the Arima-HiC v2 kit. In brief, 20–50 mg of frozen tissue (stored at –80 °C) was fixed, and the DNA crosslinked using a TC buffer with 22% formaldehyde concentration (final concentration 2%). After crosslinking, the tissue was homogenised using the Diagnocine Power Masher-II and BioMasher-II tubes and pestles. Following the Arima-HiC v2 kit manufacturer's instructions, crosslinked DNA was digested using a restriction enzyme master mix. The 5’-overhangs were filled in and labelled with biotinylated nucleotides and proximally ligated. An overnight incubation was carried out for enzymes to digest remaining proteins and for crosslinks to reverse. A clean up was performed with SPRIselect beads prior to library preparation. Additionally, the biotinylation percentage was estimated using the Qubit Fluorometer v4.0 (Thermo Fisher Scientific) and Qubit HS Assay Kit and Arima-HiC v2 QC beads.

### Library preparation and sequencing

Library preparation and sequencing were performed at the WSI Scientific Operations core.

***PacBio HiFi***

At a minimum, samples were required to have an average fragment size exceeding 8 kb and a total mass over 400 ng to proceed to the low input SMRTbell Prep Kit 3.0 protocol (Pacific Biosciences, California, USA), depending on genome size and sequencing depth required. Libraries were prepared using the SMRTbell Prep Kit 3.0 (Pacific Biosciences, California, USA) as per the manufacturer's instructions. The kit includes the reagents required for end repair/A-tailing, adapter ligation, post-ligation SMRTbell bead cleanup, and nuclease treatment. Following the manufacturer’s instructions, size selection and clean up was carried out using diluted AMPure PB beads (Pacific Biosciences, California, USA). DNA concentration was quantified using the Qubit Fluorometer v4.0 (Thermo Fisher Scientific) with Qubit 1X dsDNA HS assay kit and the final library fragment size analysis was carried out using the Agilent Femto Pulse Automated Pulsed Field CE Instrument (Agilent Technologies) and gDNA 55kb BAC analysis kit.

Samples were sequenced using the Sequel IIe system (Pacific Biosciences, California, USA). The concentration of the library loaded onto the Sequel IIe was in the range 40–135 pM. The SMRT link software, a PacBio web-based end-to-end workflow manager, was used to set-up and monitor the run, as well as perform primary and secondary analysis of the data upon completion.

***Hi-C***

For Hi-C library preparation, DNA was fragmented using the Covaris E220 sonicator (Covaris) and size selected using SPRISelect beads to 400 to 600 bp. The DNA was then enriched using the Arima-HiC v2 kit Enrichment beads. Using the NEBNext Ultra II DNA Library Prep Kit (New England Biolabs) for end repair, A-tailing, and adapter ligation. This uses a custom protocol which resembles the standard NEBNext Ultra II DNA Library Prep protocol but where library preparation occurs while DNA is bound to the Enrichment beads. For library amplification, 10 to 16 PCR cycles were required, determined by the sample biotinylation percentage. The Hi-C sequencing was performed using paired-end sequencing with a read length of 150 bp on an Illumina NovaSeq 6000 instrument.

***RNA***

Poly(A) RNA-Seq libraries were constructed using the NEB Ultra II RNA Library Prep kit, following the manufacturer’s instructions. RNA sequencing was performed on the Illumina NovaSeq X instrument.

### Genome assembly, curation and evaluation

***Assembly***

The original assembly of HiFi reads was performed using Hifiasm ( Cheng *et al.*, 2021) with the --primary option. Haplotypic duplications were identified and removed with purge_dups ( Guan *et al.*, 2020). Hi-C reads are further mapped with bwa-mem2 ( Vasimuddin *et al.*, 2019) to the primary contigs, which are further scaffolded using the provided Hi-C data ( Rao *et al.*, 2014) in YaHS ( Zhou *et al.*, 2023) using the --break option. Scaffolded assemblies are evaluated using Gfastats ( Formenti *et al.*, 2022), BUSCO ( Manni *et al.*, 2021) and MERQURY.FK ( Rhie *et al.*, 2020).

The mitochondrial genome was assembled using MitoHiFi ( Uliano-Silva *et al.*, 2023), which runs MitoFinder ( Allio *et al.*, 2020) and uses these annotations to select the final mitochondrial contig and to ensure the general quality of the sequence.

***Assembly curation***

The assembly was decontaminated using the Assembly Screen for Cobionts and Contaminants (ASCC) pipeline (article in preparation). Manual curation was primarily conducted using PretextView ( Harry, 2022), with additional insights provided by JBrowse2 ( Diesh *et al.*, 2023) and HiGlass ( Kerpedjiev *et al.*, 2018). Scaffolds were visually inspected and corrected as described by Howe *et al*. (2021). Any identified contamination, missed joins, and mis-joins were corrected, and duplicate sequences were tagged and removed. entire process is documented at https://gitlab.com/wtsi-grit/rapid-curation (article in preparation).

***Assembly quality assessment***

The Merqury.FK tool ( Rhie *et al.*, 2020), run in a Singularity container ( Kurtzer *et al.*, 2017), was used to evaluate *k*-mer completeness and assembly quality for the primary and alternate haplotypes using the *k*-mer databases ( *k* = 31) that were computed prior to genome assembly. The analysis outputs included assembly QV scores and completeness statistics.

A Hi-C contact map was produced for the final version of the assembly. The Hi-C reads were aligned using bwa-mem2 ( Vasimuddin *et al.*, 2019) and the alignment files were combined using SAMtools ( Danecek *et al.*, 2021). The Hi-C alignments were converted into a contact map using BEDTools ( Quinlan & Hall, 2010) and the Cooler tool suite ( Abdennur & Mirny, 2020). The contact map was visualised in HiGlass ( Kerpedjiev *et al.*, 2018).

The genome was analysed within the BlobToolKit environment ( Challis *et al.*, 2020) and BUSCO scores ( Manni *et al.*, 2021) were calculated.

Table 4 contains a list of relevant software tool versions and sources.

**Table 4.** Software tools: versions and sources.

Software tool	Version	Source
BlobToolKit	4.2.1	https://github.com/blobtoolkit/blobtoolkit
BUSCO	5.3.2	https://gitlab.com/ezlab/busco
Hifiasm	0.16.1-r375	https://github.com/chhylp123/hifiasm
HiGlass	1.11.6	https://github.com/higlass/higlass
Merqury	MerquryFK	https://github.com/thegenemyers/MERQURY.FK
MitoHiFi	2	https://github.com/marcelauliano/MitoHiFi
PretextView	0.2	https://github.com/wtsi-hpag/PretextView
purge_dups	1.2.3	https://github.com/dfguan/purge_dups
YaHS	yahs-1.1.91eebc2	https://github.com/c-zhou/yahs

### Genome annotation

The BRAKER2 pipeline ( Brůna *et al.*, 2021) was used in the default protein mode to generate annotation for the *Philonthus spinipes* assembly (GCA_963082785.1) in Ensembl Rapid Release at the European Bioinformatics Institute (EBI).

### Wellcome Sanger Institute – Legal and Governance

The materials that have contributed to this genome note have been supplied by a Darwin Tree of Life Partner. The submission of materials by a Darwin Tree of Life Partner is subject to the **‘Darwin Tree of Life Project Sampling Code of Practice’**, which can be found in full on the Darwin Tree of Life website here. By agreeing with and signing up to the Sampling Code of Practice, the Darwin Tree of Life Partner agrees they will meet the legal and ethical requirements and standards set out within this document in respect of all samples acquired for, and supplied to, the Darwin Tree of Life Project.

Further, the Wellcome Sanger Institute employs a process whereby due diligence is carried out proportionate to the nature of the materials themselves, and the circumstances under which they have been/are to be collected and provided for use. The purpose of this is to address and mitigate any potential legal and/or ethical implications of receipt and use of the materials as part of the research project, and to ensure that in doing so we align with best practice wherever possible. The overarching areas of consideration are:

•   Ethical review of provenance and sourcing of the material

•   Legality of collection, transfer and use (national and international)

Each transfer of samples is further undertaken according to a Research Collaboration Agreement or Material Transfer Agreement entered into by the Darwin Tree of Life Partner, Genome Research Limited (operating as the Wellcome Sanger Institute), and in some circumstances other Darwin Tree of Life collaborators.

## Data Availability

European Nucleotide Archive: Philonthus spinipes. Accession number PRJEB61909; https://identifiers.org/ena.embl/PRJEB61909. The genome sequence is released openly for reuse. The *Philonthus spinipes* genome sequencing initiative is part of the Darwin Tree of Life (DToL) project. All raw sequence data and the assembly have been deposited in INSDC databases. Raw data and assembly accession identifiers are reported in Table 1 and Table 2.

## Author information

Members of the University of Oxford and Wytham Woods Genome Acquisition Lab are listed here: https://doi.org/10.5281/zenodo.12157525.

Members of the Darwin Tree of Life Barcoding collective are listed here: https://doi.org/10.5281/zenodo.12158331

Members of the Wellcome Sanger Institute Tree of Life Management, Samples and Laboratory team are listed here: https://doi.org/10.5281/zenodo.12162482.

Members of Wellcome Sanger Institute Scientific Operations: Sequencing Operations are listed here: https://doi.org/10.5281/zenodo.12165051.

Members of the Wellcome Sanger Institute Tree of Life Core Informatics team are listed here: https://doi.org/10.5281/zenodo.12160324.

Members of the Tree of Life Core Informatics collective are listed here: https://doi.org/10.5281/zenodo.12205391.

Members of the Darwin Tree of Life Consortium are listed here: https://doi.org/10.5281/zenodo.4783558.
