# Supplementary material for: The genome sequence of a rove beetle, Philonthus spinipes Sharp, 1874
Source: Wellcome Open Res. 2025 Apr 25;10:217. [Version 1] doi: 10.12688/wellcomeopenres.23996.1 (PMC12134740; doi:10.12688/wellcomeopenres.23996.1)
Supplement: Peer review history

# Reviewer report: Reviewer response for version 1

**DOI:** 10.21956/wellcomeopenres.26473.r123725

**Published:** 2025-06-03

**Authors:** Chenyang Cai

**Affiliations:** 1 Chinese Academy of Sciences, Nanjing, China

This study presents a high-quality chromosomal-level genome assembly for the rove beetle *Philonthus spinipes*, a valuable contribution to coleopteran genomics. The assembly leverages state-of-the-art methods—PacBio HiFi long reads (37× coverage) and Hi-C scaffolding (153.41 Gb data)—to scaffold 671.1 Mb into 25 chromosomal pseudomolecules, including the X chromosome, and includes a complete mitochondrial genome (19.08 kb). Gene annotation identified 30,004 protein-coding genes.

Are sufficient details of methods and materials provided to allow replication by others?

Yes

Is the rationale for creating the dataset(s) clearly described?

Yes

Are the datasets clearly presented in a useable and accessible format?

Yes

Are the protocols appropriate and is the work technically sound?

Yes

Reviewer Expertise:

beetles

I confirm that I have read this submission and believe that I have an appropriate level of expertise to confirm that it is of an acceptable scientific standard.

# Reviewer report: Reviewer response for version 1

**DOI:** 10.21956/wellcomeopenres.26473.r122855

**Published:** 2025-05-27

**Authors:** Joseph Parker

**Affiliations:** 1 California Institute of Technology, Pasadena, California, USA

The paper presents a long read assembly of *Philonthus spinipes*. The assembly quality is high with 99.9% of the genome in 25 pseudomolecules and a BUSCO >99%. However, there are some areas in which the paper could be improved:

 1. The background to the study is weak. The paper could benefit from putting the genome in the context of rove beetle biology/biodiversity and other rove beetle genomes that have been sequenced. There is no rationale for the current study beyond Darwin ToL.

 2. RNA for the transcriptome was extracted from the abdomen only and so the gene counts supported by RNAseq data will be an underestimate, missing genes expressed in other tissues. This should be acknowledged in the main text of the paper.

 3. Assignment of the X chromosome based on synteny to a leaf beetle seems like a strange rationale when there are other sequenced rove beetles out there, as well as Tribolium where the X is definitively identified. A figure to support this synteny would be valuable.

Are sufficient details of methods and materials provided to allow replication by others?

Yes

Is the rationale for creating the dataset(s) clearly described?

Partly

Are the datasets clearly presented in a useable and accessible format?

Yes

Are the protocols appropriate and is the work technically sound?

Partly

Reviewer Expertise:

entomology, evolution

I confirm that I have read this submission and believe that I have an appropriate level of expertise to confirm that it is of an acceptable scientific standard, however I have significant reservations, as outlined above.

# Reviewer report: Reviewer response for version 1

**DOI:** 10.21956/wellcomeopenres.26473.r123723

**Published:** 2025-05-26

**Authors:** Henrique Antoniolli

**Affiliations:** 1 Universidade Federal do Rio Grande do Sul (UFRGS), Porto Alegre, Brazil

In this study, Mann et al. provide a highly contiguous genome assembly for a rove beetle, *Philonthus spinipes*. The methods employed are in line with current sequencing technology and are well described. All data produced are accessible on online databases.

 Minor issues:

- The mitochondrial genome should also be annotated and submitted to appropriate sections in online databases.
- Introduction is too concise and does not state what motivated the sequencing of its genome.

Are sufficient details of methods and materials provided to allow replication by others?

Yes

Is the rationale for creating the dataset(s) clearly described?

No

Are the datasets clearly presented in a useable and accessible format?

Yes

Are the protocols appropriate and is the work technically sound?

Yes

Reviewer Expertise:

Genomics, evolution

I confirm that I have read this submission and believe that I have an appropriate level of expertise to confirm that it is of an acceptable scientific standard, however I have significant reservations, as outlined above.
